# Therapeutic vaccination following early antiretroviral therapy elicits highly functional T cell responses against conserved HIV-1 regions

**DOI:** 10.1038/s41598-023-42888-3

**Published:** 2023-10-11

**Authors:** Jakub Kopycinski, Hongbing Yang, Gemma Hancock, Matthew Pace, Ellen Kim, John Frater, Wolfgang Stöhr, Tomás Hanke, Sarah Fidler, Lucy Dorrell, RIVER trial study group

**Affiliations:** 1 Nuffield Department of Medicine, University of Oxford, Oxford, UK; 2 Medical Research Council Clinical Trials Unit, University College London, London, UK; 3 Joint Research Centre for Human Retrovirus Infection, Kumamoto University, Kumamoto, Japan; 4 Department of Infectious Disease, Imperial College London, and National Institute for Health Research Imperial Biomedical Research Centre, London, UK; 5 Immunocore Ltd, 93 Park Drive, Milton Park, Abingdon, OX14 4RY Oxon UK

**Keywords:** Viral infection, Vaccines, Immune cell isolation

## Abstract

‘Kick and kill’ cure strategies aim to induce HIV protein expression in latently infected cells (kick), and thus trigger their elimination by cytolytic T cells (kill). In the Research in Viral Eradication of HIV Reservoirs trial (NCT02336074), people diagnosed with primary HIV infection received immediate antiretroviral therapy (ART) and were randomised 24 weeks later to either a latency-reversing agent, vorinostat, together with ChAdV63.HIVconsv and MVA.HIVconsv vaccines, or ART alone. This intervention conferred no reduction in HIV-1 reservoir size over ART alone, despite boosting virus-specific CD4+ and CD8+ T cells. The effects of the intervention were examined at the cellular level in the two trial arms using unbiased computational analysis of polyfunctional scores. This showed that the frequency and polyfunctionality of virus-specific CD4+ and CD8+ T cell populations were significantly increased over 12 weeks post-vaccination, compared to the ART-only arm. HIV-specific IL-2-secreting CD8+ T cells also expanded significantly in the intervention arm and were correlated with antiviral activity against heterologous HIV in vitro. Therapeutic vaccination during ART commenced in primary infection can induce functional T cell responses that are phenotypically similar to those of HIV controllers. Analytical therapy interruption may help determine their ability to control HIV in vivo.

## Introduction

Antiretroviral therapy (ART) has dramatically improved survival for people living with HIV (PLWH) but alone is insufficient to cure HIV-1 (hereafter referred to as HIV) infection. Additional interventions are needed to eliminate long-lived CD4+ cells harbouring quiescent replication-competent proviruses, which are responsible for virological relapse if ART is interrupted^1^. HIV-specific T cell responses curtail primary viremia and modulate the provirus landscape during ART, therefore, therapeutic vaccination has been extensively explored as a strategy to amplify these responses, with the aim of purging reservoirs^2,3^. However, few therapeutic vaccine regimens have provided clinical benefit, likely reflecting both limitations in the design of HIV immunogens and underlying immunological impairment. Initiation of ART during primary HIV infection (PHI) mitigates immune damage, restricts the formation of reservoirs and increases the potential to achieve post-treatment control (PTC)^4,5^. Rationally designed immunogens have therefore been tested in the context of early ART, either alone or to deliver a ‘kick and kill’, a strategy in which a latency-reversing agent is used to ‘kick’ HIV antigen expression, with vaccine-induced CD8+ T cells delivering the ‘kill’. Results have been mixed. Chimpanzee adenovirus (ChAdV63) and modified vaccinia Ankara (MVA) vectors encoding an HIV conserved immunogen comprising the 14 most conserved sub-protein regions of HIV (ChAdV63.HIVconsv + MVA.HIVconsv) were administered together with romidepsin, a histone deacetylase inhibitor (HDACi) in the BCN01/02 studies^6–8^. Nearly a quarter of participants showed PTC for 32 weeks during an analytical therapy interruption (ATI). Furthermore, vaccination with a ChAdV63- / MVA-vectored immunogen targeting regions of vulnerability within the HIV proteome was associated with PTC for up to 22 weeks in a subset of participants^9^. By contrast, vaccination with human adenovirus type 26 (Ad26) and MVA vectors encoding HIV mosaic immunogens did not lead to control of viremia after ATI^10^.

In the Research in Viral Eradication of HIV Reservoirs (RIVER) trial, the first randomised placebo-controlled study of a kick and kill strategy, individuals who had initiated ART shortly after a diagnosis of PHI and had received at least 24 weeks’ continuous therapy were randomly allocated to an intervention arm involving administration of ChAdV63.HIVconsv and MVA.HIVconsv vaccines together with the HDACi, vorinostat (ART + V + V) or placebo (ART-only). The intervention did not reduce HIV DNA or RNA in CD4+ T cells, despite a significant amplification of HIVconsv-specific CD4+ and CD8+ T cell responses^11^. These results led us to investigate the functionality of expanded HIV-specific T cells.

## Results

The RIVER study design has been described previously and is summarised in Methods^11^. In brief, participants started ART within 1 month of a confirmed diagnosis of PHI and were treated for at least 24 weeks at the time of randomisation. Provided that plasma HIV RNA was < 50 copies/ml, participants were randomised 1:1 to receive either ART + V + V or ART alone for a further 18 weeks. The ChAdV63.HIVconsv vaccine (5 × 10^10^ vp) was administered on the day of randomisation and MVA.HIVconsv (2 × 10^8^ pfu) at week 8 post-randomisation. Vorinostat (400 mg) was administered every 3 days during post-randomisation weeks 0–4, up to a total of 10 doses. Sample availability permitting, HIVconsv-specific T cell responses were evaluated at the following time-points: enrolment (pre-ART and close to the diagnosis of PHI if enrolled with a prospective diagnosis, ie.stratum 1 participants only, see Methods), randomisation (ie. pre-vaccination), week 9 and week 12 post-randomisation (ie. 1 week and 4 weeks after MVA.HIVconsv vaccination, and denoted PR-W9 and PR-W12 respectively). The baseline characteristics of the participants who were included in this analysis are summarised in Table 1.

**Table 1:** RIVER participant characteristics at enrolment and randomisation.

	ART + V + V	ART only
Subjects evaluated (n)	27^a^	24^a^
CD4 count^a^, cells/µl—median (IQR)		
Screening	560 (480–708)	517 (436–575)
Randomisation	716 (611–786)	660 (552–854)
PR-W8 + 3	742 (497–836)	730 (538–900)
CD4/CD8 ratio^b^—median (IQR)		
Screening	0.74 (0.56–1.26)	0.61 (0.38–0.82)
Randomisation	1.08 (0.92–1.49)	1.07 (0.75–1.25)
PR-W8 + 3	1.17 (0.87–1.44)	1.08 (0.78–1.41)
HIV RNA at screening, log_10_ copies/ml—median (IQR)	4.57 (2.7–6.3)	4.8 (3.5–5.6)
HIV RNA at randomisation^c^, copies/ml—median (IQR)	< 20 (< 20– < 40)	< 20 (< 20– < 40)
Days from PHI diagnosis to ART initiation—median (IQR)	17 (9–23)	14 (3–23)

^a^Data shown in table reflect participants with samples available for analysis at enrolment.

^b^CD4 counts were measured at screening, randomisation and PR-W8, day 3; these were the closest time-points to the visits when HIV-specific T cell responses were analysed.

^c^At randomisation, all participants but one had HIV RNA values below the lower limit of detection (< 20 copies/ml for Taqman-2, < 40 copies/ml for other assays).

### Polyfunctionality of HIV-specific CD4+ T cell responses is increased following therapeutic vaccination

**Figure 1:**
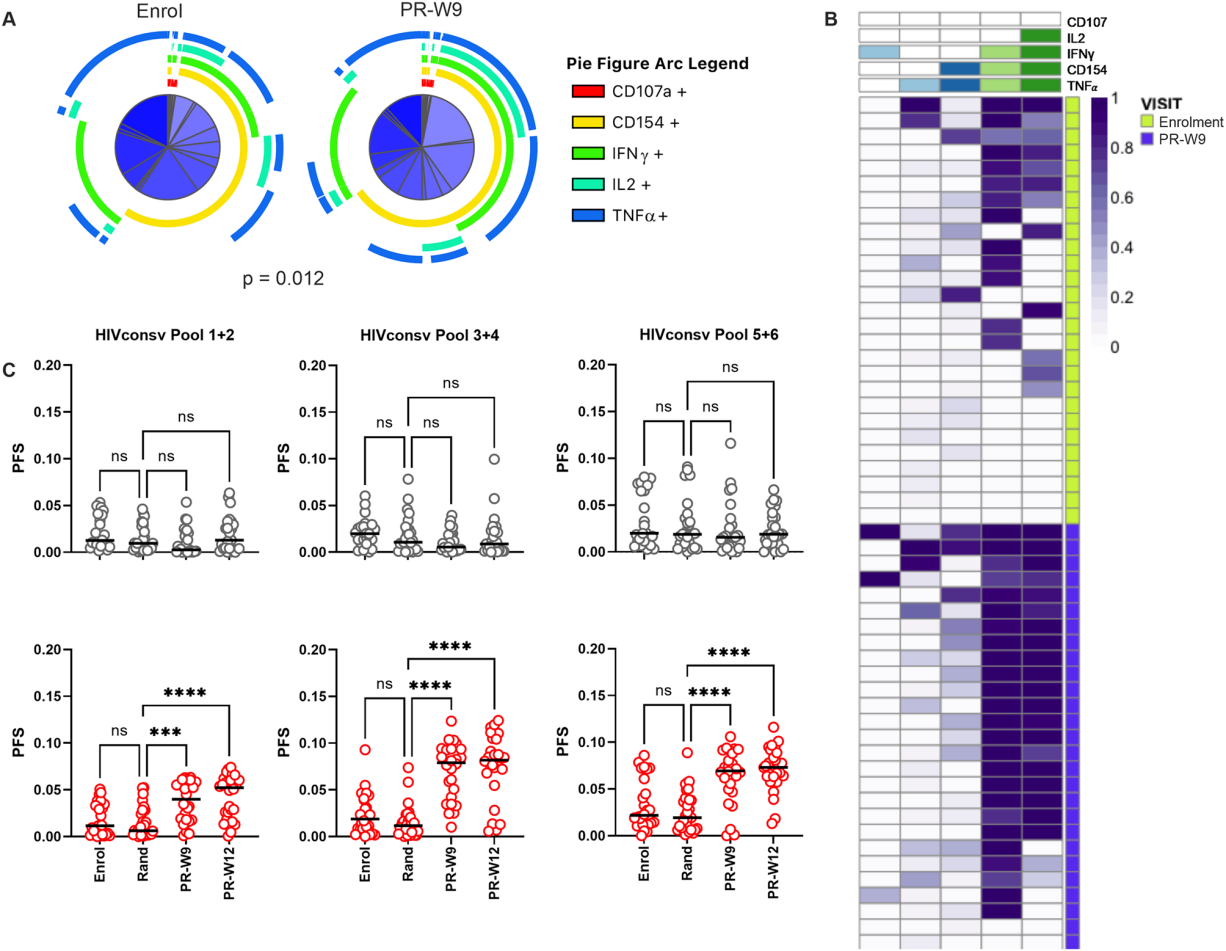
Polyfunctionality of HIV-specific CD4 + T cell responses is increased following therapeutic vaccination. (**A**) Pie charts show the functional phenotype of aggregated, mock-subtracted HIVconsv-specific CD4 + T cells of RIVER participants in the ART + V + V arm at enrolment and post-randomisation week 9 (PR-W9). Permutation test was performed to assess overall difference in response phenotypes. Pie slices are further defined in Supplementary Fig. 1. (**B**) Representative heat-plot generated using COMPASS shows response phenotypes of ART + V + V participants at enrolment and PR-W9, in CD4 + T cell sub-populations specific for HIVconsv peptide Pool 3 + 4 that exceeded a pre-specified threshold of 5 events. Columns correspond to these functional subsets modelled by COMPASS. Rows correspond to participants. The key to the columns is indicated by the blue and green bars: from left to right—1-functional IFN-γ, 1- functional TNF-α, 2-functional CD154 / TNF-α, 3-functional CD154 / IFN-γ / TNF-α, 4-functional CD154 / IFN-γ / TNF-α / IL-2. Each cell of the heatmap shows the probability (represented by colour intensity) that the corresponding functional subset has an antigen-specific response as defined by the key. (**C**) Dot plots show longitudinal polyfunctional score (PFS) of aggregated HIVconsv-specific CD4 + T cell responses in (top,  symbols) ART-only (n = 27) and (bottom,  symbols) ART + V + V (n = 30) arms. Left—Pool 1 + 2; middle—Pool 3 + 4; right—Pool 5 + 6. Horizontal bars indicate medians. Statistical significance was determined by repeated measures mixed effects model with Sidak’s multiple comparisons test. *p < 0.05, **p < 0.01, ***p < 0.001, ****p < 0.0001.

We have previously reported that participants in the ART + V + V arm showed a significant increase in the frequency of HIVconsv-specific CD4+ T cells that co-expressed CD154, a marker of T cell activation, and IFN-γ (median 15.1-fold; 0.0064 to 0.097% CD4+ T cells) from randomisation to week 9 post-randomisation (PR-W9)^11^. To further investigate the functional phenotype of the responding cells, we first analysed the HIVconsv-specific T cell populations for expression of combinations of CD107a, CD154, IFN-γ, TNF-α and IL-2, at enrolment and at PR-W9. This enabled a comparison of T cell populations that were primed by HIV infection with those that were induced or boosted by therapeutic vaccination. Simplified Presentation of Incredibly Complex Evaluations (SPICE) analysis showed a significant difference in the responding phenotypes at these two time-points (Permutation test, *p* = 0.012; Fig. 1A). The largest difference was seen in CD4+ T cells displaying the CD154+ /IFN-γ + /IL2+ /TNF-α + phenotype, which were significantly expanded at PR-W9 (Wilcoxon rank sum test, *p* = 0.001, Supplementary Figs. 1 and 2). While ART-only participants also had detectable HIVconsv-specific CD4+ T cells at enrolment, their phenotype as determined by SPICE was unchanged at PR-W9 (data not shown)^11^.

Polyfunctionality of HIVconsv-specific T cells responding was next assessed using COMPASS, a Bayesian hierarchical framework algorithm that allows the definition of a functional score (FS) and polyfunctionality score (PFS); the latter provides summary of functionality after weighting according to the individual functionalities and relative dominance of the sub-populations of cells within the defined parental (CD8+ or CD4 +) responder population^12^. The posterior probabilities of antigen-specific responses across all functional subsets were assessed for each arm of the study at all visits up to PR-W12. Responses were resolved to antigenic sub-regions within the HIVconsv immunogen using pools of overlapping peptides. PFS were significantly higher post-randomisation in the ART + V + V arm for each peptide pool tested (repeated measures mixed effects model with Sidak’s multiple comparison test; Fig. 1B and C). This was due primarily to higher frequencies of 3- or 4-functional CD4+ cells, chiefly expressing CD154, IFN-γ, TNF-α and IL-2 (Supplementary Fig. 2). By contrast, PFS did not change significantly over time in the ART-only arm (Fig. 1C).

### Polyfunctionality of HIV-specific CD8+ T cell responses is increased following therapeutic vaccination

**Figure 2:**
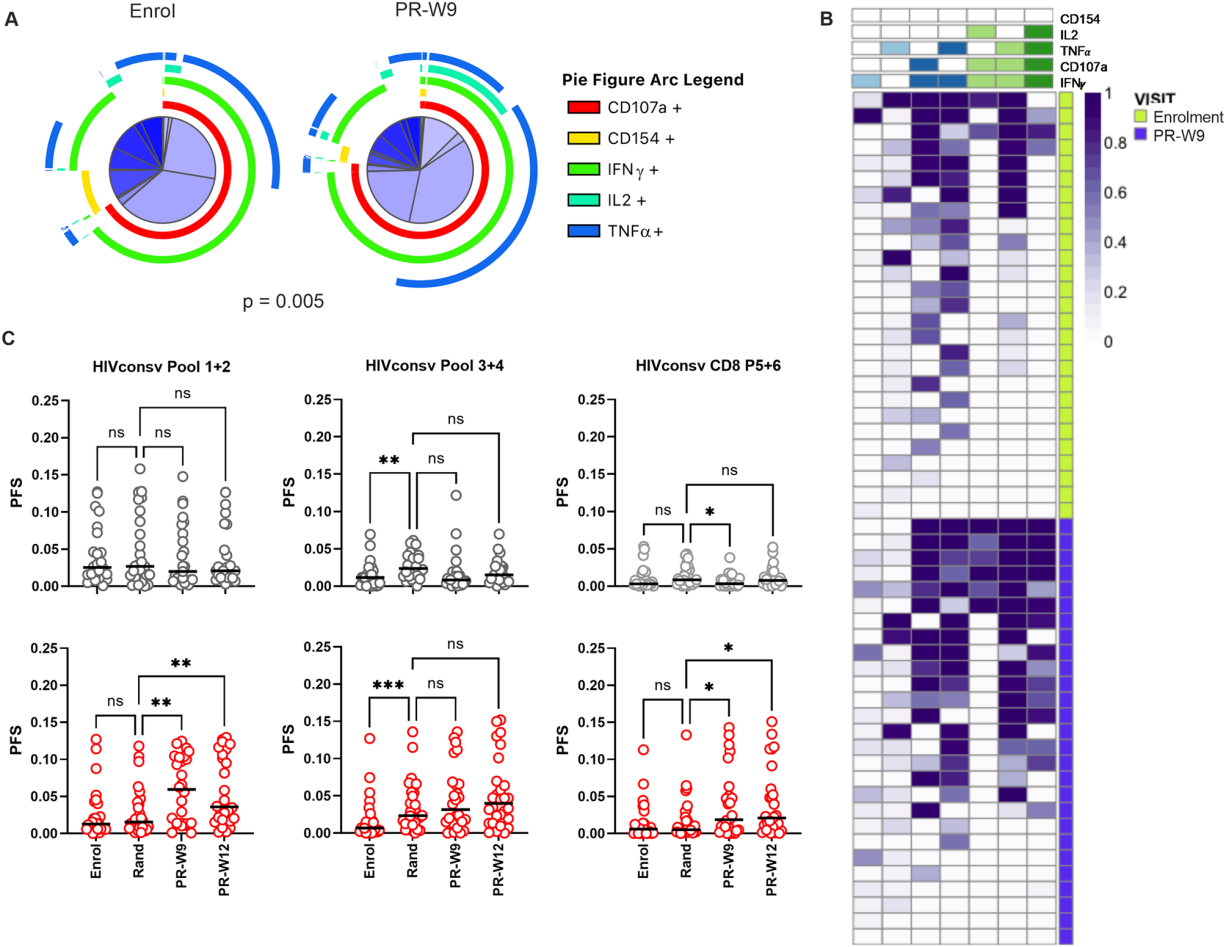
Polyfunctionality of HIV-specific CD8 + T cell responses is increased following therapeutic vaccination. (**A**) Pie charts show the functional phenotype of aggregated, mock-subtracted HIVconsv-specific CD8 T-cells of HIV-infected individuals pre- and post-vaccination; overall difference in response phenotypes was assessed by permutation test. Pie slices are further defined in Supplementary Fig. 3. (**B**) Representative heat-plot generated using COMPASS shows response phenotypes of ART + V + V participants at enrolment and PR-W9, in CD8 + T cell sub-populations specific for HIVconsv peptide Pool 3 + 4 that exceeded a pre-specified threshold of 5 events. Columns correspond to these functional subsets modelled by COMPASS. Rows correspond to participants. The key to the columns is indicated by the blue and green bars: from left to right—1-functional IFN-γ, 1- functional TNFα, 2-functional CD107a / IFN-γ, 2-functional IFN-γ / TNF-α, 3-functional IFN-γ / CD107a / Il-2, 3-functional IFN-γ / TNF-α / Il-2, 4-functional CD107a / IFN-γ / TNF-α / IL-2. Each cell of the heatmap shows the probability (represented by colour intensity) that the corresponding functional subset has an antigen-specific response as defined by the key. (**C**) Dot plots show longitudinal PFS of aggregated HIVconsv-specific CD8 + T cell responses in (top,  symbols) ART-only (n = 27) and (bottom,  symbols) ART + V + V arms (n = 30). Left—Pool 1 + 2; middle—Pool 3 + 4; right—Pool 5 + 6. Horizontal bars indicate medians. Statistical significance was determined by repeated measures mixed effects model with Sidak’s multiple comparisons test. *p < 0.05, **p < 0.01.

The majority of participants in both study arms had detectable HIV-specific CD107+ IFN-γ + CD8+ T cell responses at enrolment or randomisation, as shown by Fidler et al.^11^; these were also amplified in the ART + V + V arm following vaccination, with a > threefold increase in median frequency between randomisation and PR-W9 (0.0518–0.194% CD8+ T cells). To determine whether vaccine-expanded responses were qualitatively different from those primed by HIV, we assessed the functional phenotype of CD8+ T cells using the same methodology as for CD4+ T cells. Nine weeks post-randomisation, the overall CD8+ T cell response phenotype had changed significantly in the ART + V + V arm (PR-W9 vs. enrolment; Permutation test, *p* = 0.005; Fig. 2A). Vaccinated individuals showed an increase in the frequency of several sub-populations, most of which co-expressed CD107a and IFN-γ in combination with either of TNF-α and IL2, with the increase in the 4-functional CD107a+ /IFN-γ+ /IL2+ /TNF-α + population being most significant (Wilcoxon Rank Sum test, *p* = 0.0001; Supplementary Figs. 3 and 4). These changes were corroborated by COMPASS analysis of PFS (Fig. 2B). The differences between pre- and post-randomisation visits were strongest for responses to Pools 1 + 2 and 5 + 6, with ART + V + V recipients showing an increased proportion of CD107a+ /IFN-γ + CD8+ T cells that co-expressed IL-2, while ART-only recipients showed no change in the responses to Pools 1–4 and a slight decrease in responses to Pool 5 + 6 (Fig. 2B and C). We excluded the possibility that ART + V + V recipients had higher frequencies of HIVconsv-specific CD8+ IFN-γ + TNF-α + cells prior to randomisation (they were marginally higher in the ART-only arm than the ART + V + V arm at enrolment; Wilcoxon Rank sum test, *p* = 0.045) or more polyfunctional CD8 + T cells at this time-point (Permutation test for overall phenotype difference between study arms, *p* = 0.6; Supplementary Fig. 5).

### Polyfunctionality of CD4+ and CD8+ subsets of HIVconsv-specific T cells pre-and post-randomisation are strongly correlated

**Figure 3:**
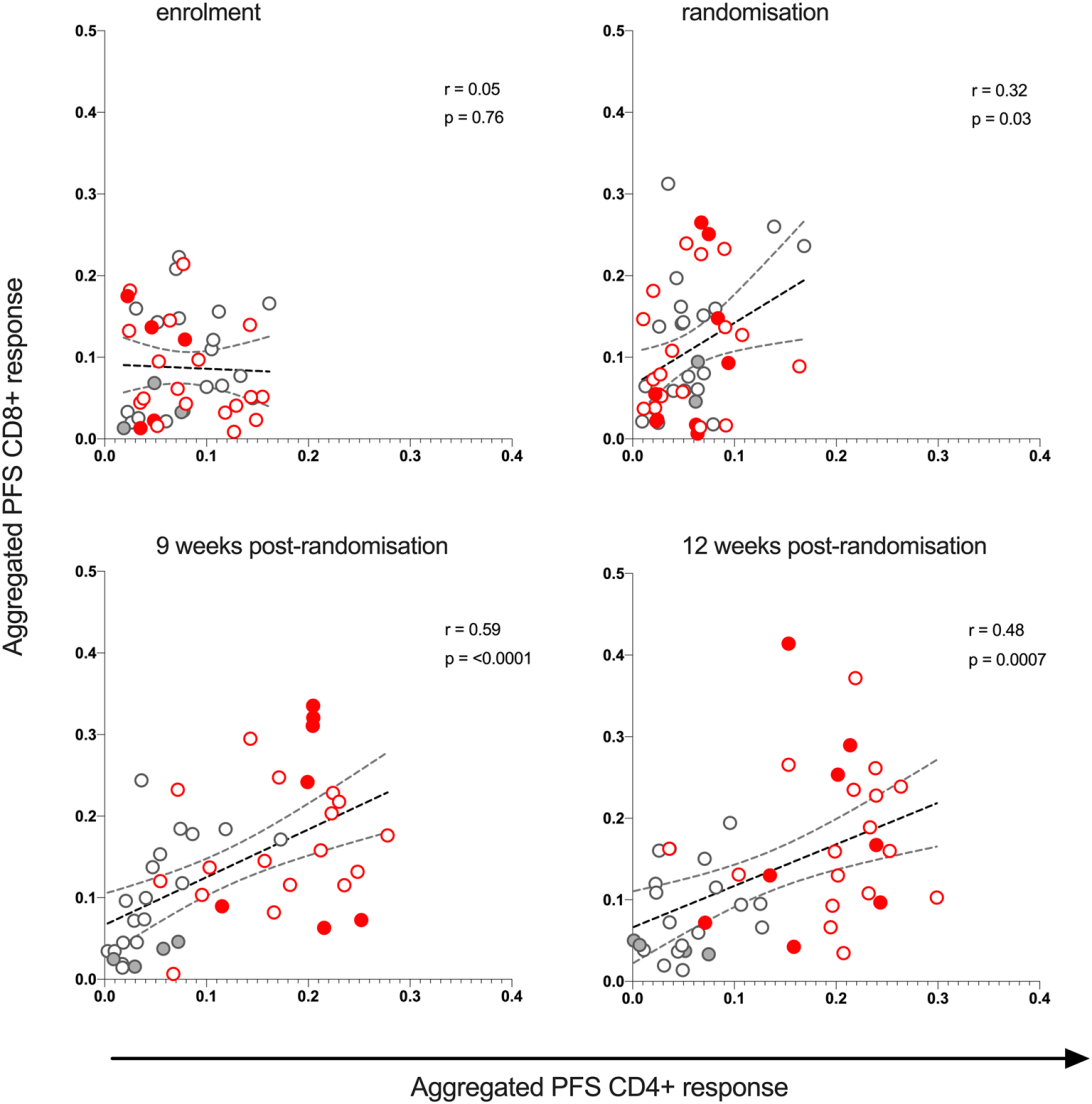
Correlation between PFS of HIVconsv-specific CD4 + and CD8 + T cell populations pre-and post-randomisation. Scatter plots show matched, aggregated PFS of HIVconsv-specific CD4 + and CD8 + T cell populations at enrolment, randomisation PR-W9 and PR-W12.  symbols—ART-only participants;  symbols—ART + V + V participants. Participants with HLA-B*27:05, HLA-B*57:01 and HLA-B*58:01 alleles are indicated by filled circles ( and  symbols), to show their distribution across treatment arms. Correlation was determined from Spearman’s rho coefficient.

Next, a possible correlation between PFS for HIVconsv-specific CD4+ and CD8+ T cells was explored. ART-only and ART + V + V participants formed distinct clusters after randomisation, consistent with an increase in PFS in the latter group (Fig. 3). The PFS for total HIVconsv-specific CD4+ and CD8+ T cells were positively correlated at randomisation, PR-W9 and PR-W12 but not at enrolment, with the strongest association being observed at PR-W9 (r = 0.59, *p* < 0.0001; Fig. 3). Of note, median CD4/CD8 ratio was > 1 from randomisation onwards but < 1 at screening (Table 1).

### CD8+ T cell antiviral activity in vaccinees is positively associated with the frequency of HIVconsv-specific CD8+ T cells expressing IL-2

**Figure 4:**
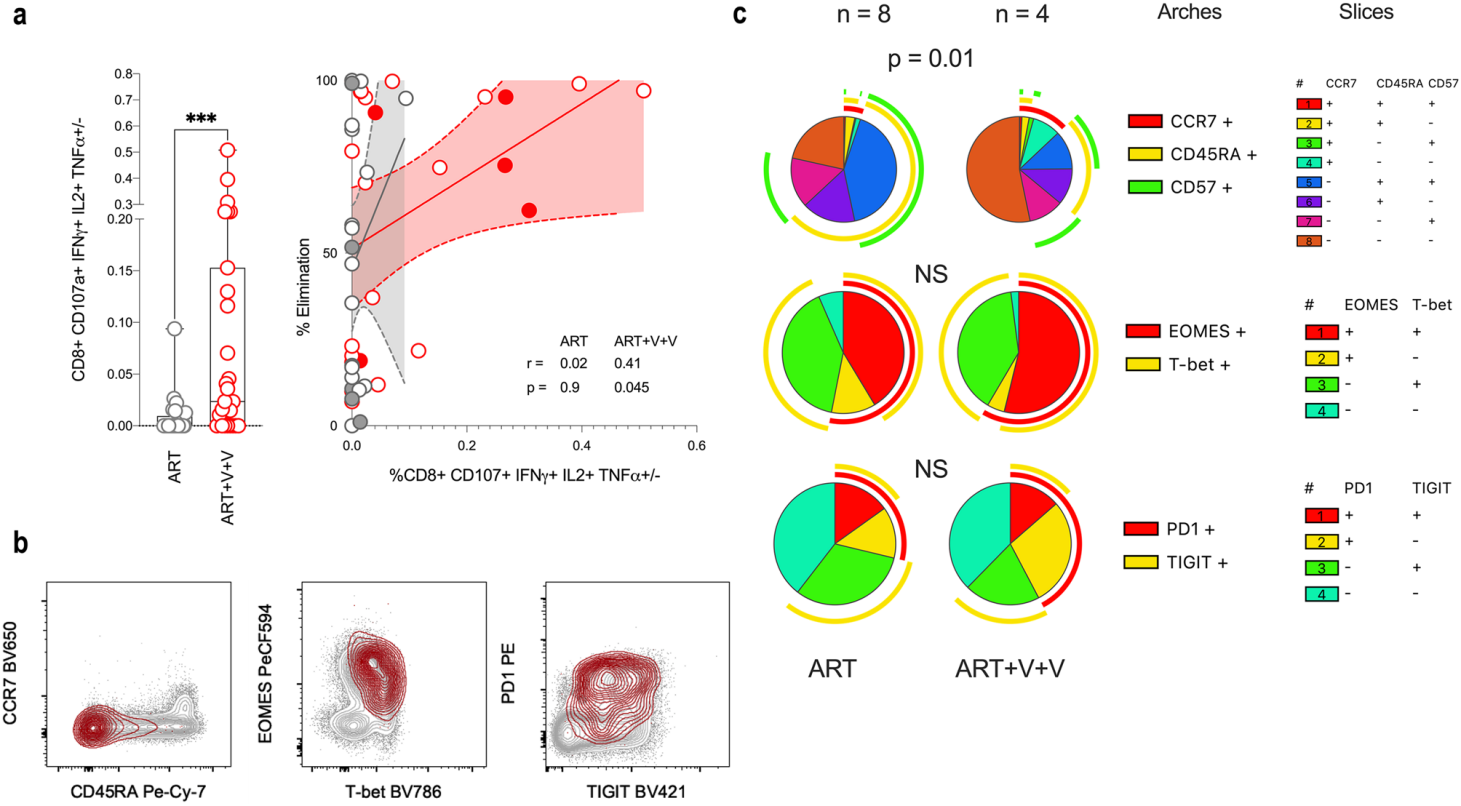
Frequencies of HIVconsv-specific CD8 + T cells expressing IL-2 correlate with CD8 + T cell antiviral activity in vaccinees but not ART-only recipients. (**A**) Dot plot (left) shows frequencies of HIVconsv-specific CD8 + T-cells in ART + V + V participants (, n = 23) compared with the ART-only group (, n = 24). Difference was assessed by Mann–Whitney test. Scatter plot (right) shows correlation between frequencies of CD107a + IFN-γ + IL-2 + TNF-α + /- cells and CD8 + T cell-mediated infected cell elimination, determined by Spearman’s rho coefficient. Participants with HLA-B*27, HLA-B*57:01, HLA-B*58:01 are indicated by  and  symbols. (**B**) Representative flow cytometry plots overlaying HIVconsv-specific (IFN-γ +) populations (red) onto parental CD8 + T cell populations (grey) in the context of expression of memory markers CCR7 and CD45RA, transcription factors T-bet and EOMES, and activation / exhaustion markers PD-1 and TIGIT. (**C**) SPICE plots showing the composition of HIVconsv-specific IFN-γ + T cell populations in ART-only and ART + V + V participants (those with available samples in which there were > 10 IFN-γ + events were included). Differences between the treatment arms were assessed by permutation tests (Supplementary Fig. 6).

The primary analysis of the RIVER trial showed that CD8+ T cell antiviral activity, as indicated by the capacity to eliminate HIV-infected CD4+ T cells in vitro, waned over time in ART-only recipients yet was preserved in ART + V + V recipients at PR-W9 and PR-W12^11^. Several possible explanations were considered. Certain HLA class I alleles have been previously been associated with potent antiviral activity (HLA-B*27:05, HLA-B*57:01 and B*58:01)^13,14^. RIVER participants were not significantly enriched for these alleles in the ART + V + V arm (Fisher’s exact test, *p* = 0.7)^15^. Furthermore, antiviral activity was not associated with superior polyfunctionality scores per se, as defined by the PFS of aggregated HIVconsv-specific CD4+ and CD8+ T cell populations, at pre- or post-randomisation time points in either study arm (Data available on request). On the other hand, HIVconsv-specific 3- or 4-functional CD8+ T cells that expressed IL-2 were significantly elevated post-randomisation in vaccinees and were found to be positively correlated with infected cell elimination (r = 0.41, *p* = 0.045; Fig. 4A).

To rule out the possibility that antiviral activity was affected by preferential expansion of specific effector cell subsets in the ART + V + V arm, or progression to a state of exhaustion in the ART-only arm, we characterised HIVconsv-specific CD8+ T cells post-randomisation according to several markers of antigen experience (Fig. 4B and C; Supplementary Fig. 6). Despite the difference in frequency of HIVconsv-specific CD8+ T cells between the two study arms, effector memory cells (CD45RA^-^ CCR7^-^) were the dominant sub-population in both. Terminally differentiated effectors (CD45RA^+^ CCR7^-^ CD57^+/-^) were preferentially expanded in ART-only participants whereas the proportion of central memory cells (CD45RA^-^ CCR7^+^) was significantly greater in ART + V + V participants. The groups did not differ with respect to the EOMES / T-bet ratio nor the expression of PD-1 or TIGIT in HIVconsv-specific CD8+ T cells.

## Discussion

Polyfunctional virus-specific CD4+ and CD8+ T cells that secrete IL-2 and are capable of lysing HIV-infected cells in vitro are typically detected in HIV controllers and rarely observed in chronic HIV infection, even after prolonged ART^14,16–18^. This study shows that therapeutic vaccination in people with HIV who receive very early ART can elicit HIV-specific T cell responses with a similar phenotype to those observed in HIV controllers, exemplified by sustained cytokine-secreting and cytolytic functions and by the establishment of a central memory pool^19^. The positive association between PFS for CD4+ and CD8+ T cells from randomisation through PR-W12 indicates that control of viremia facilitates the maintenance of CD4+ and CD8+ T cell functionality to a similar extent. Moreover, the increased CD4+ and CD8+ T cell PFS to HIVconsv Pool 1 + 2, which covers Gag sequences within HIVconsv, suggest that Gag-specific T cell functionality was enhanced by vaccination. We have shown previously that CD8+ T cell targeting of functionally constrained regions within Gag proteins is a major driver of CD8+ T cell antiviral activity^14,20^. We surmise, therefore, that the superior CD8+ T cell antiviral effect in the ART + V + V arm was due to a combination of re-focusing, ie. higher frequencies of functional Gag-specific CD8+ T cells, and of HIV-specific IL-2-secreting CD4+ and CD8+ T cells, since IL-2 is essential for HIV-specific T cell proliferation and survival^21,22^. Taken together, the results presented here indicate that a fully functional immune response to conserved viral epitopes was elicited by the vaccination strategy. Other factors must therefore underlie the apparent lack of impact of the study intervention on viral reservoirs in RIVER trial participants. For example, the reservoir in early treated PLWH is very low to begin with and therefore to achieve any further statistically significant decrease is extremely challenging^4,23^. It has been noted previously that vorinostat administration had minimal effect on viral reactivation in this study, therefore, it is possible that cellular reservoirs were not sensitised to cytolytic T cells following vaccination or not accessed by the vaccine-elicited T cell effectors.

In contrast to the BCN01/02 studies, which evaluated the ChAdV63.HIVconsv and MVA.HIVconsv vaccines together with the HDACi, romidepsin in a similar protocol, the RIVER trial did not include an ATI, therefore, we do not know whether the responses induced in this study would have been sufficient to control recrudescent virus and delay rebound. PTC has hitherto been observed infrequently after therapeutic vaccination. Colby et al. observed a delay of < 1 week after administration of HIV mosaic immunogens in a prime-boost strategy, and it is noteworthy that the vaccination regimen in this study enhanced the functionality of Pol- and Env-, but not Gag-specific T cells, possibly as a consequence of antigenic competition and lack of re-focusing towards vulnerable regions of the viral proteome^10,24^. We and others have shown previously that viral load set-point after PHI is strongly influenced by CD8+ T cell responses to the most functionally constrained regions within the HIV proteome, the majority of which are found within Gag proteins^14,25^. This may explain the longer period of PTC observed following vaccination strategies targeted to such regions^9^. Nevertheless, the lack of sustained PTC observed with most therapeutic vaccines tested to date raises the question of whether vaccine-induced or boosted T cells traffic to or access tissue reservoirs, including sanctuary sites such as B cell follicles, and whether anamnestic responses elicited by these vaccines may be too slow to eliminate infected cells immediately upon viral reactivation^8,26,27^. If so, then alternative approaches will be needed for effective targeting of viral reservoirs. Access to B cell follicles may require treatment with IL-15 or an IL-15 superagonist^28,29^. Adoptive T cell therapies or T cell redirecting agents such as bispecific T cell engagers may be better able to overcome the temporal and spatial barriers that limit the effectiveness of vaccine-boosted HIV-specific T cells^30^.

Accumulating evidence supports initiation of ART as early as possible after HIV acquisition, in order to prevent seeding of immune-privileged sites and acute inflammation, which drives the destruction of lymphoid architecture, early loss of CD4+ T cell help and progressive T cell dysfunction^31–33^. Our data provide evidence to suggest that early ART enables the generation of highly functional virus-specific T cells; however, this cannot be definitely established due to the lack of a comparator arm comprising vaccinees who initiated ART during the chronic phase of infection. Nevertheless, early ART may not only increase the potential for immune-based therapies to achieve PTC but may also ensure that PLWH can be adequately protected by vaccination against diverse infectious pathogens. This has been highlighted recently by Frater et al. who showed that ART-experienced people living with HIV were capable of mounting functional humoral and cellular responses to SARS-CoV-2 vaccination that were equivalent to those in people without HIV^34^.

## Methods

### Study approval

All individuals gave written informed consent to participate in the RIVER study (NCT02336074), a phase 2, open­label randomised controlled trial that was conducted at 6 clinical sites in the UK during December 2015 to November 2017. Approval for the study was obtained from the South Central—Oxford A Research Ethics Committee, UK (reference: 14/SC/1372) from all participating centres in accordance with the principles of the Declaration of Helsinki.

### Study participants

Participants aged 18–60 years who had acquired HIV infection within the preceding ≤ 6 months and had started ART within 1 month from confirmed PHI diagnosis were enrolled in the RIVER trial (NCT02336074) if they fulfilled pre-specified recent infection criteria. Eligible consenting participants were recruited either to stratum 1 (ART initiation at enrolment and randomisation 24 weeks later) or stratum 2 (previously initiated on ART within 4 weeks of a PHI diagnosis, with ART duration of at least 6 months and up to 2 years after PHI). Sixty men were randomly assigned to receive ART + V + V or ART alone. Full details, including baseline characteristics for the entire study population, have been reported previously^11^. CD4+ cell counts, CD4/CD8 ratio and plasma viral load were measured at screening, randomisation and post-randomisation week 8, day 3 (PR-W8 + 3). These were the closest time-points to those visits when the HIVconsv-specific T cell analyses were performed, therefore, the evaluated participants’ results at these time-points are shown in Table 1.

Peripheral blood mononuclear cells (PBMC) were obtained from the RIVER study participants and cryopreserved immediately. For each participant, cryopreserved PBMC from all study visits were thawed and analysed simultaneously. For the analyses described here, samples from the randomisation visit were available in 27 participants in the ART-only arm, and 30 participants in the ART + V + V arm. Samples were missing from other time-points in 2–3 participants in the ART-only arm and 2–4 participants in the ART + V + V arm.

### Intracellular cytokine staining (ICS)

Cryopreserved PBMC were thawed, washed and rested overnight in RPMI medium supplemented with 10% fetal calf serum (FCS), 1% (v/v) penicillin/streptomycin and 2mM L-glutamine (R10 medium) at 37 °C, then stimulated with peptide pools (15-mers with 11aa overlap) corresponding to the HIVconsv vaccine transgene; 2 μg/ml), mock control (0.45% DMSO) and positive controls (SEB, 5 μg/ml; CMV pp65, 2 μg/ml, NIH AIDS Reagent Repository) at 37 °C for 6 h in the presence of Golgiplug, Golgistop (BD Biosciences) and CD107a BV421^35^. Following viability and surface staining, cells were fixed using BD cytofix/cytoperm solution according to the manufacturer's instructions and intracellularly stained for multiparameter flow cytometric analysis using reagents as listed in Supplementary Table 1. At least 10,000 viable singlet CD3^+^ CD4^+^ and CD8^+^ lymphocyte events were acquired using a BD Fortessa X20 cytometer.

### Multiparameter flow cytometry analysis

Initial analysis was performed using FlowJo v9.9.7. T cell functionality was analysed using COMPASS^12^. The polyfunctional score (PFS) for HIVconsv-specific responses was assessed using COMbinatorial Polyfunctionality analysis of Antigen-Specific T-cell Subsets, an open-sourced platform employing Bayesian hierarchical frameworks to model observed functional cell subsets and select those most likely to be antigen-specific responses^12^. The PFS weighs the different responding subsets by their degree of functionality and the posterior probabilities were reported in both CD4+ and CD8+ T cell subsets expressing combinations of IFN-γ, IL2, TNF-α, CD107a and CD154. Analysis of cell surface phenotype and transcriptional profile was performed using PESTLE and SPICE^36^. Individuals with HIVconsv-specific T cell CD8+ responses (> 10 IFN-γ  + CD107a + events within the CD3+ CD8+ population) were selected for further analysis using a panel of antibodies to the following: CCR7, CD45RA, CD57, PD-1, TIGIT, Eomesodermin (EOMES) and T-bet (Supplementary Table 1).

### CD8+ T cell infected cell elimination assay

CD8+ T cell antiviral activity was assessed in an infected cell elimination assay, as previously described^11^. Briefly, cryopreserved PBMC were thawed and expanded using anti-human CD3 (OKT3) antibody at 100ng/ml (BD Pharmingen) and IL-2 at 100 IU/ml (Proleukin) for 7 days. CD4+ and CD8+ populations were subsequently purified by positive selection with immunomagnetic beads (MACS, Miltenyi Biotec) according to the manufacturer’s instructions. CD4+ T cells were super-infected with HIV IIIB (National Institute for Biological Standards and Control, United Kingdom) by spinoculation at a multiplicity of infection (MOI) of 0.01 and cultured alone or together with autologous CD8+ T cells at CD4:CD8 ratios of 1:1 or 10:1 for 7 days. On day 7, cells from replicate wells were pooled and stained for viability, CD3, CD4, CD8 and intracellular Gag p24^37^. Samples were acquired on a Fortessa X20 cytometer (BD) and analysed by FlowJo v9.9.6. Assay acceptance required acquisition of at least 2000 viable CD3+ /CD8- lymphocyte events. Reduction in p24 + cells was expressed as percent infected cell elimination and determined as follows: [(fraction of p24 + cells in CD4 + T cells cultured alone) − (fraction of p24 + in CD4+ T cells cultured with CD8+ cells)]/(fraction of p24 + cells in CD4+ T cells cultured alone) × 100.

### Statistical analysis

HIVconsv peptide pool-specific PFS in T cell populations was assessed longitudinally using repeated measures mixed effects model with Sidak’s test for multiple comparisons. Correlations were assessed using simple linear regression analysis. SPICE 6 was used to determine statistically significant differences in T cell phenotypes using permutation tests, t tests and Wilcoxon rank sum tests. All other statistical analyses were performed using GraphPad Prism v9.1.2.

### Supplementary Information

Supplementary Information.

## Data Availability

The data supporting the results in this manuscript are provided within the main figures and supplementary material or, in the case of polyfunctional scores for aggregated T cell populations, will be made available on request. Please contact: jakub.kopycinski@vaccitech.co.uk.

## References

[1] Chun TW (1997). Presence of an inducible HIV-1 latent reservoir during highly active antiretroviral therapy. Proc. Natl. Acad. Sci. U. S. A..

[2] Pitman MC, Lau JSY, McMahon JH, Lewin SR (2018). Barriers and strategies to achieve a cure for HIV. Lancet HIV.

[3] Warren JA (2020). The HIV-1 latent reservoir is largely sensitive to circulating T cells. Elife.

[4] Ananworanich J (2016). HIV DNA set point is rapidly established in acute HIV infection and dramatically reduced by early ART. EBioMedicine.

[5] Namazi G (2018). The control of HIV after antiretroviral medication pause (CHAMP) study: Posttreatment controllers identified from 14 clinical studies. J. Infect. Dis..

[6] Letourneau S (2007). Design and pre-clinical evaluation of a universal HIV-1 vaccine. PLoS One.

[7] Mothe B (2019). Therapeutic vaccination refocuses T-cell responses towards conserved regions of HIV-1 in early treated individuals (BCN 01 study). EClinicalMedicine.

[8] Mothe B (2020). HIVconsv vaccines and romidepsin in early-treated HIV-1-infected individuals: Safety, immunogenicity and effect on the viral reservoir (Study BCN02). Front. Immunol..

[9] Bailón L (2022). Safety, immunogenicity and effect on viral rebound of HTI vaccines in early treated HIV-1 infection: A randomized, placebo-controlled phase 1 trial. Nat. Med..

[10] Colby DJ (2020). Safety and immunogenicity of Ad26 and MVA vaccines in acutely treated HIV and effect on viral rebound after antiretroviral therapy interruption. Nat. Med..

[11] Fidler S (2020). Antiretroviral therapy alone versus antiretroviral therapy with a kick and kill approach, on measures of the HIV reservoir in participants with recent HIV infection (the RIVER trial): A phase 2, randomised trial. Lancet (London, England).

[12] Lin L (2015). COMPASS identifies T-cell subsets correlated with clinical outcomes. Nat. Biotechnol..

[13] Saez-Cirion A (2007). HIV controllers exhibit potent CD8 T cell capacity to suppress HIV infection ex vivo and peculiar cytotoxic T lymphocyte activation phenotype. Proc. Natl. Acad. Sci. U. S. A..

[14] Hancock G (2015). Identification of effective subdominant anti-HIV-1 CD8+ T cells within entire post-infection and post-vaccination immune responses. PLoS Pathog..

[15] Migueles SA (2011). Trivalent adenovirus type 5 HIV recombinant vaccine primes for modest cytotoxic capacity that is greatest in humans with protective HLA class I alleles. PLoS Pathog..

[16] Emu B (2008). HLA class I-restricted T-cell responses may contribute to the control of human immunodeficiency virus infection, but such responses are not always necessary for long-term virus control. J. Virol..

[17] Betts MR (2006). HIV nonprogressors preferentially maintain highly functional HIV-specific CD8+ T cells. Blood.

[18] Migueles SA (2009). Defective human immunodeficiency virus-specific CD8+ T-cell polyfunctionality, proliferation, and cytotoxicity are not restored by antiretroviral therapy. J. Virol..

[19] Burgers WA (2009). Association of HIV-specific and total CD8+ T memory phenotypes in subtype C HIV-1 infection with viral set point. J. Immunol..

[20] Yang H (2021). Incoming HIV virion-derived Gag spacer peptide 2 (p1) is a target of effective CD8(+) T cell antiviral responses. Cell Rep..

[21] Younes SA (2003). HIV-1 viremia prevents the establishment of interleukin 2-producing HIV-specific memory CD4+ T cells endowed with proliferative capacity. J. Exp. Med..

[22] Trautmann L (2012). Profound metabolic, functional, and cytolytic differences characterize HIV-specific CD8 T cells in primary and chronic HIV infection. Blood.

[23] Leyre L (2020). Abundant HIV-infected cells in blood and tissues are rapidly cleared upon ART initiation during acute HIV infection. Sci. Transl. Med..

[24] Kallas EG (2019). Antigenic competition in CD4(+) T cell responses in a randomized, multicenter, double-blind clinical HIV vaccine trial. Sci. Transl. Med..

[25] Mothe B (2011). Definition of the viral targets of protective HIV-1-specific T cell responses. J. Transl. Med..

[26] Banga R (2016). PD-1(+) and follicular helper T cells are responsible for persistent HIV-1 transcription in treated aviremic individuals. Nat. Med..

[27] Hansen SG (2011). Profound early control of highly pathogenic SIV by an effector memory T-cell vaccine. Nature.

[28] Watson DC (2018). Treatment with native heterodimeric IL-15 increases cytotoxic lymphocytes and reduces SHIV RNA in lymph nodes. PLoS Pathog..

[29] Webb GM (2018). The human IL-15 superagonist ALT-803 directs SIV-specific CD8(+) T cells into B-cell follicles. Blood Adv..

[30] Yang H, Wallace Z, Dorrell L (2018). Therapeutic targeting of HIV reservoirs: How to give T cells a new direction. Front. Immunol..

[31] Macías J (2001). Structural normalization of the lymphoid tissue in asymptomatic HIV-infected patients after 48 weeks of potent antiretroviral therapy. AIDS.

[32] Schuetz A (2014). Initiation of ART during early acute HIV infection preserves mucosal Th17 function and reverses HIV-related immune activation. PLoS Pathog..

[33] Jain V (2013). Antiretroviral therapy initiated within 6 months of HIV infection is associated with lower T-cell activation and smaller HIV reservoir size. J. Infect. Dis..

[34] Frater J (2021). Safety and immunogenicity of the ChAdOx1 nCoV-19 (AZD1222) vaccine against SARS-CoV-2 in HIV infection: A single-arm substudy of a phase 2/3 clinical trial. Lancet HIV.

[35] Borthwick N (2014). Vaccine-elicited human T cells recognizing conserved protein regions inhibit HIV-1. Mol. Ther..

[36] Roederer M, Nozzi JL, Nason MC (2011). SPICE: Exploration and analysis of post-cytometric complex multivariate datasets. Cytometry A.

[37] Yang H (2013). Improved quantification of HIV-1-infected CD4+ T cells using an optimised method of intracellular HIV-1 gag p24 antigen detection. J. Immunol. Methods.

