# Supplementary material for: Therapeutic vaccination following early antiretroviral therapy elicits highly functional T cell responses against conserved HIV-1 regions
Source: Sci Rep. 2023 Oct 11;13:17155. doi: 10.1038/s41598-023-42888-3 (PMC10567821; doi:10.1038/s41598-023-42888-3)
Supplement: Supplementary file 1 — Supplementary Information. [file 41598_2023_42888_MOESM1_ESM.pdf]

Supplementary Material for: *Therapeutic vaccination following early antiretroviral therapy elicits highly functional T cell responses against conserved HIV-1 regions*

Jakub Kopycinski[1], Hongbing Yang[1], Gemma Hancock[1], Matthew Pace[1], Ellen Kim[1], John Frater[1], Wolfgang Stöhr[2], Tomás Hanke[1,3], Sarah Fidler[4], Lucy Dorrell[1,5], RIVER trial study group

RIVER trial study group members: Eric Sandström, Janet Darbyshire, Frank Post, Christopher Conlon, Jane Anderson, Mala Maini, Timothy Peto, Peter Sasieni, Veronica Miller, Ian Weller, Sarah Fidler, John Frater, Abdel Babiker, Wolfgang Stöhr, Sarah Pett, Lucy Dorrell, Matthew Pace, Natalia Olejniczak, Helen Brown, Nicola Robinson, Jakub Kopycinski, Hongbing Yang, Tomáš Hanke, Alison Crook, Steven Kaye, Myra McClure, Otto Erlwein, Andrew Lovell, Maryam Khan, Michelle Gabrielle, Rachel Bennett, Aminata Sy, Adam Gregory, Fleur Hudson, Charlotte Russell, Gemma Wood, Hanna Box, Cherry Kingsley, Katie Topping, Andrew Lever, Mark Wills, Axel Fun, Mikaila Bandara, Damian Kelly, Simon Collins, Alex Markham, Mary Rauchenberger, Yinka Sowunmi, Shaadi Shidfar, Dominic Hague, Mark Nelson, Maddalena Cerrone, Nadia Castrillo Martinez, Tristan Barber, Alexandra Schoolmeesters, Christine Weaver, Orla Thunder, Jane Rowlands, Christopher Higgs, Serge Fedele, Margherita Bracchi, Lervina Thomas, Peter Bourke, Nneka Nwokolo, Gaynor Lawrenson, Marzia Fiorino, Hinal Lukha, Sabine Kinloch-de Loes, Margaret Johnson, Alice Nightingale, Nnenna Ngwu, Patrick Byrne, Zoe Cuthbertson, Martin Jones, Tina Fernandez, Amanda Clarke, Martin Fisher, Rebecca Gleig, Vittorio Trevitt, Colin Fitzpatrick, Tanya Adams, Fiounnuala Finnerty, John Thornhill, Heather Lewis, Kristin Kuldanek, Julie Fox, Julianne Lwanga, Hiromi Uzu, Ming Lee, Simon Merle, Patrick O'Rourke, Isabel Jendrulek, Taras ZarkoFlynn, Mark Taylor, Juan Manuel Tiraboschi, Tammy Murray

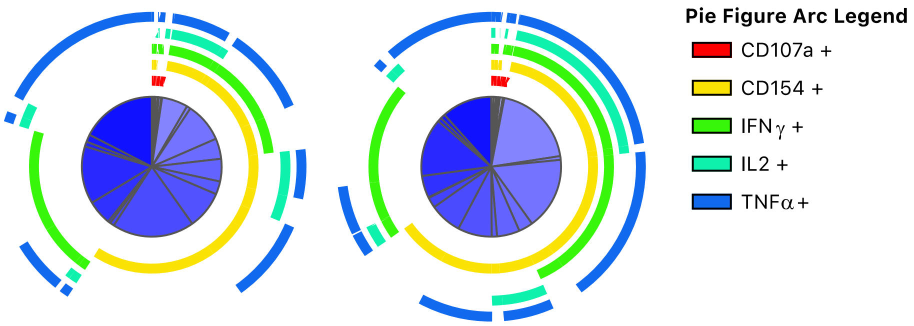

ART+V+V arm: HIVconsv-specific CD4+ T cells						
CD107a	CD154	IFN-γ	IL2	TNF-α	Enrolment vs PR-W9	
+	+	+	+	+	0.1	1
+	+	+	+	-	1.0	2
+	+	+	-	+	0.2	3
+	+	+	-	-	0.8	4
+	+	-	+	+	0.1	5
+	+	-	+	-	1.0	6
+	+	-	-	+	0.8	7
+	+	-	-	-	0.5	8
+	-	+	+	+	0.3	9
+	-	+	+	-	1.0	10
+	-	+	-	+	0.071	11
+	-	+	-	-	0.4	12
+	-	-	+	+	1.0	13
+	-	-	+	-	0.8	14
+	-	-	-	+	1.0	15
-	+	+	+	+	0.000	16
-	+	+	+	-	0.6	17
-	+	+	-	+	0.001	18
-	+	+	-	-	0.3	19
-	+	-	+	+	0.1	20
-	+	-	+	-	0.6	21
-	+	-	-	+	0.1	22
-	+	-	-	-	0.5	23
-	-	+	+	+	0.005	24
-	-	+	+	-	0.5	25
-	-	+	-	+	0.4	26
-	-	+	-	-	0.7	27
-	-	-	+	+	0.4	28
-	-	-	+	-	0.4	29
-	-	-	-	+	0.8	30

Supplementary Figure 1 Composition of HIVconsv-specific CD4+ T cells determined by 5-functional flow analysis in ART+V+V recipients

(A) Comparison between overall HIVconsv-specific CD4+ T cell response phenotypes at enrolment (left pie) and Week 9 post-randomisation (PR-W9, right pie) (each from main Figure 1) in the ART+V+V group using SPICE. (B) Comparison of individual responding cell subsets analysed by Wilcoxon rank sum test. P values are shown in right-hand column. Rows represent pie slices shown in (A).

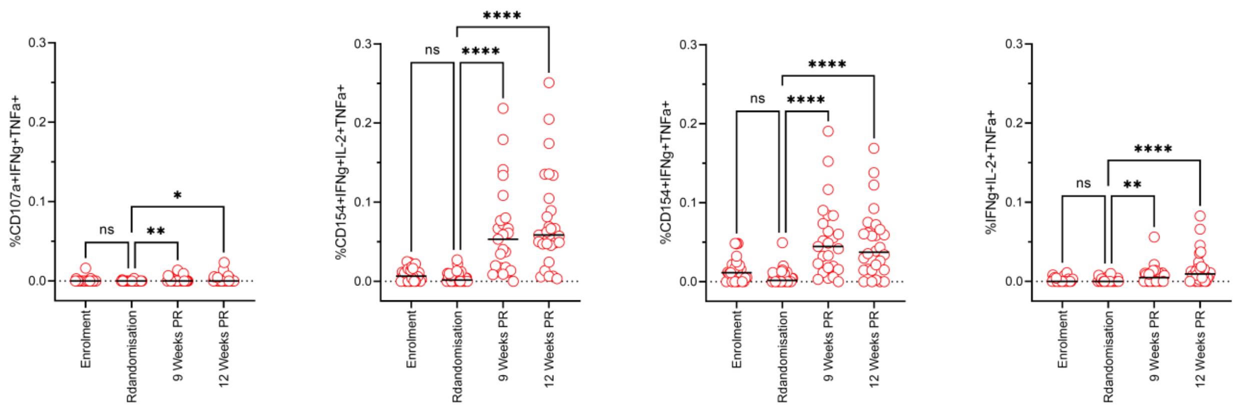

Supplementary Figure 2 Expansion of polyfunctional subpopulations within HIVconsv-specific CD4+ T cells following therapeutic vaccination

Four subpopulations were significantly expanded at PR-W9 relative to the enrolment visit (indicated by red text in the table in Supplementary Figure 1). These subpopulations were either 3-functional or 4-functional and their frequencies at each study visit are shown. Statistical significance was determined by Kruskal-Wallis test with Dunn's multiple comparisons test. *p<0.05, **p<0.01, ***p<0.001, ****p<0.0001.

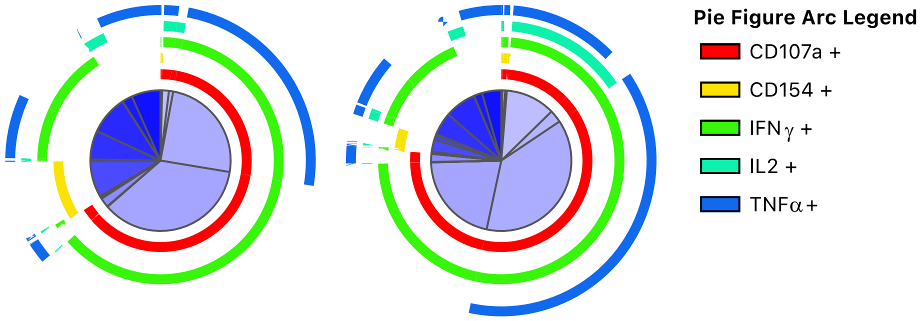

ART+V+V: HIVconsv-specific CD8+ T cell responses						
CD107a	CD154	IFN-γ	IL2	TNF-α	Enrolment vs PR-W9	
+	+	+	+	+	0.4	1
+	+	+	+	-	0.6	2
+	+	+	-	+	0.1	3
+	+	+	-	-	1.0	4
+	+	-	+	+	1.0	5
+	+	-	+	-	1.0	6
+	+	-	-	+	0.8	7
+	+	-	-	-	0.5	8
+	-	+	+	+	0.0001	9
+	-	+	+	-	0.05	10
+	-	+	-	+	0.002	11
+	-	+	-	-	0.2	12
+	-	-	+	+	0.6	13
+	-	-	+	-	0.9	14
+	-	-	-	+	0.8	15
-	+	+	+	+	0.8	16
-	+	+	+	-	0.8	17
-	+	+	-	+	0.6	18
-	+	+	-	-	0.5	19
-	+	-	+	+	1.0	20
-	+	-	+	-	0.5	21
-	+	-	-	+	0.8	22
-	+	-	-	-	1.0	23
-	-	+	+	+	0.016	24
-	-	+	+	-	0.5	25
-	-	+	-	+	0.1	26
-	-	+	-	-	0.1	27
-	-	-	+	+	1.0	28
-	-	-	+	-	0.4	29
-	-	-	-	+	0.7	30

Supplementary Figure 3 Composition of HIVconsv-specific CD8+ T cells determined by 5-functional flow analysis in ART+V+V recipients

(A) Comparison of the overall HIVconsv-specific CD8+ T cell response phenotypes in ART+V+V individuals at enrolment (left pie) and 9 weeks post-randomisation (right pie) (each from main Figure 2) using SPICE. (B) Comparison of individual responding cell subsets (Wilcoxon rank sum test). P values are shown in right-hand column. Rows represent pie slices shown in (A).

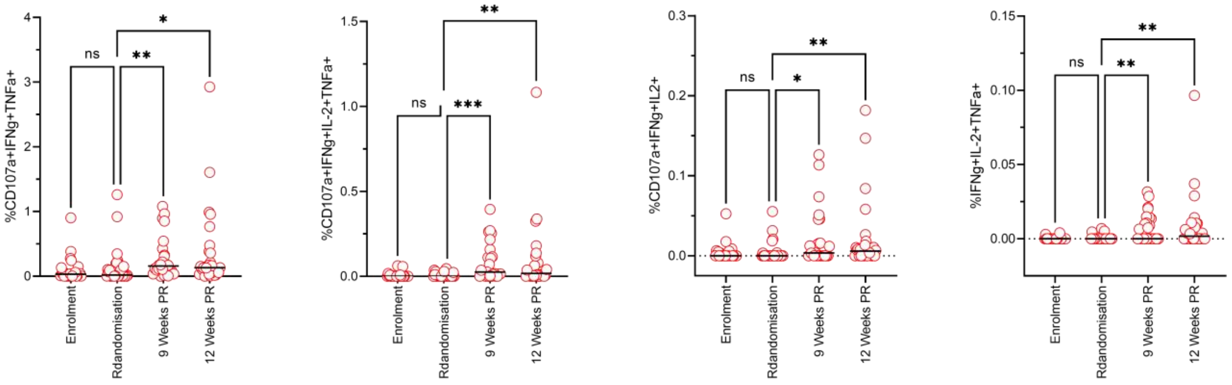

Supplementary Figure 4 Expansion of polyfunctional subpopulations within HIVconsv-specific CD8+ T cells following therapeutic vaccination

Four subpopulations were significantly expanded at PR-W9 relative to the enrolment visit (indicated by red text in the table in Supplementary Figure 2). These subpopulations were either 3-functional or 4-functional and their frequencies at each study visit are shown. Statistical significance was determined by Kruskal-Wallis test with Dunn's multiple comparisons test. *$p<0.05$, **$p<0.01$, ***$p<0.001$, ****$p<0.0001$.

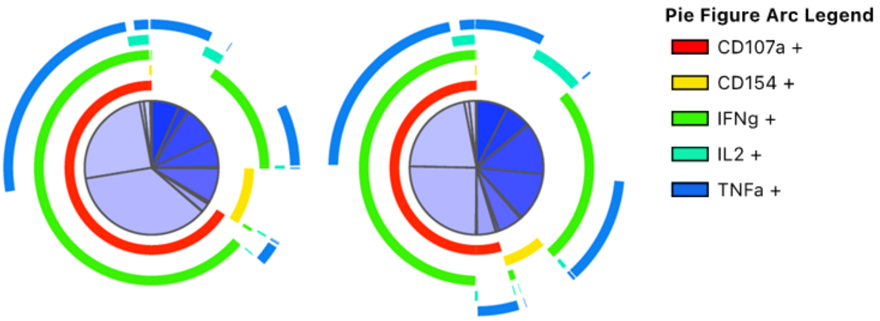

Both arms: HIVconsv-specific CD8+ T cell responses at enrolment

CD107a	CD154	IFN-γ	IL2	TNF-α	ART-only vs. ART+V+V	
+	+	+	+	+	1.0	1
+	+	+	+	-	1.0	2
+	+	+	-	+	0.8	3
+	+	+	-	-	0.9	4
+	+	-	+	+	1.0	5
+	+	-	+	-	1.0	6
+	+	-	-	+	1.0	7
+	+	-	-	-	0.5	8
+	-	+	+	+	0.2	9
+	-	+	+	-	0.5	10
+	-	+	-	+	0.4	11
+	-	+	-	-	0.5	12
+	-	-	+	+	1.0	13
+	-	-	+	-	0.7	14
+	-	-	-	+	0.1	15
-	+	+	+	+	1.0	16
-	+	+	+	-	0.4	17
-	+	+	-	+	0.8	18
-	+	+	-	-	0.8	19
-	+	-	+	+	0.7	20
-	+	-	+	-	0.9	21
-	+	-	-	+	1.0	22
-	+	-	-	-	0.5	23
-	-	+	+	+	0.9	24
-	-	+	+	-	1.0	25
-	-	+	-	+	0.045	26
-	-	+	-	-	0.1	27
-	-	-	+	+	0.6	28
-	-	-	+	-	0.3	29
-	-	-	-	+	0.8	30

Supplementary Figure 5 Comparison of HIVconsv-specific CD8+ T cell sub-populations determined by 5-functional flow analysis at enrolment, stratified by subsequent treatment allocation

(A) Comparison between overall HIVconsv-specific CD8+ T cell response phenotypes in ART-only (left pie) vs ART+V+V (right pie) arms at enrolment using SPICE. Permutation test: $p = 0.575$. (B) Comparison of individual responding cell subsets (analysed by Students t-test). P values are shown in right-hand column. Rows represent pie slices shown in (A).

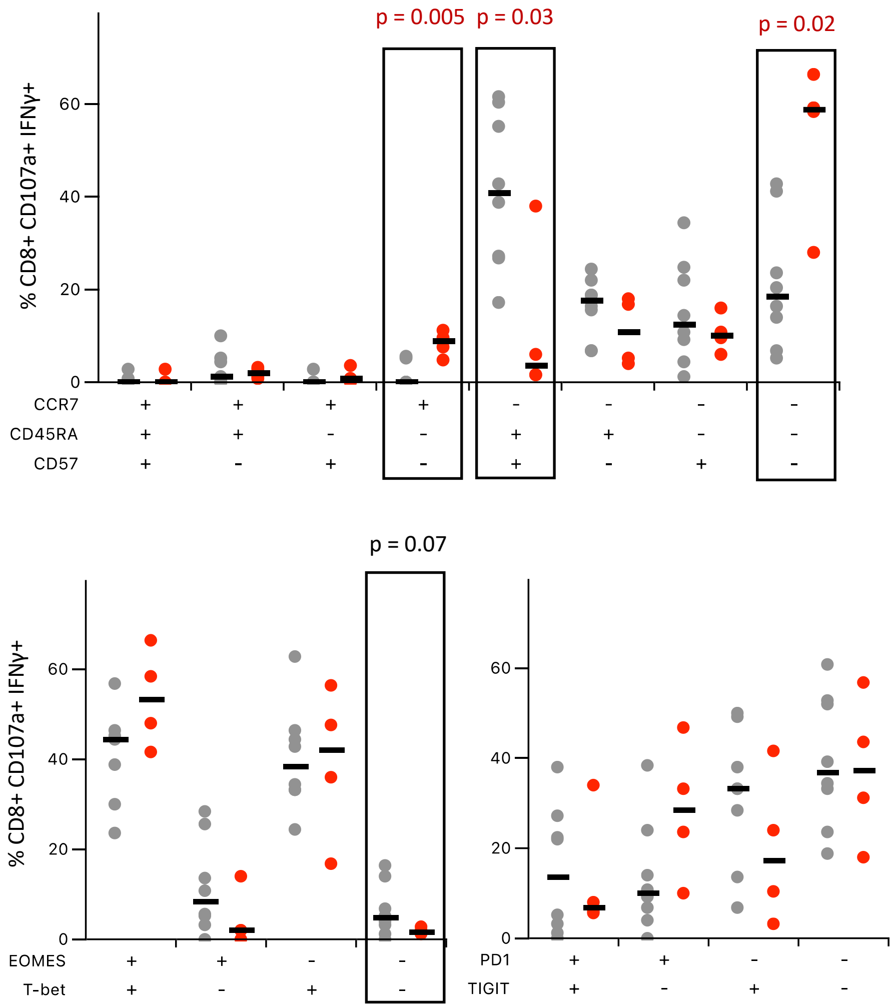

Supplementary Figure 6

Phenotypes of HIVconsv-specific IFN-γ+CD107a+ CD8+ T cell responses (>10 events): for each sub-population, individuals in ART-only (● symbols, n = 8) and ART+V+V (● symbols, n = 4) arms were compared at a post-randomisation time-point. Percentage of CD8+ IFNγ+ T cells is shown on the y-axis.

Supplementary Table 1 Reagent panels for flow cytometry analyses

Detector	Filter	Marker	Fluorochrome	Clone	Supplier	Step
Infected cell elimination panel						
V525	525/50 (505LP)	Live dead	Amcyan/V510	NA	Invitrogen	Viability
B670	670/30 (655LP)	CD4	PerCP Cy5.5	OKT4	Biolegend	Surface
V450	450/50	CD8	BV421	RPA-T8	Biolegend	
R780	780/60 (750LP)	CD3	APC “Fire”750	SK7	Biolegend	
B530	530/30 (505LP)	P24	FITC	KC57	Beckman	Intracellular
Intracellular cytokine staining panel						
V450	450/50	CD107a	BV421	H4A3	BD	
V525	525/50(505LP)	live dead	AMCyan/V510	N/A	Invitrogen	Stimulation
R780	780/60 (750LP)	CD3	APC "Fire"750	SK7	Biolegend	Viability
G610	610/20 (595LP)	CD4	Pe "Dazzle"594	RPA T4	Biolegend	Surface
B670	670/30 (655LP)	CD8	PerCP Cy5.5	RPA T8	Biolegend	
G780	780/60 (750LP)	CD154	PeCy7	24-31	Biolegend	
R670	670/30	IFNγ	APC	B27	BD	Intracellular
G586	586/15 (550LP)	IL2	PE	MQ1-17HI2	BD	
B530	530/30 (505LP)	TNFα	FITC	Mab11	BD	
CD8+ T cell Memory / Exhaustion / Transcription Factor Panel						
R780	780/60 (750LP)	CD107a	APC "Fire"750	H4A3	BD	Stimulation
V525	525/50 (505LP)	live dead	AMCyan/V510	N/A	Invitrogen	Viability
R780	730/45 (710LP)	CD3	Alexa700	SK7	Biolegend	Surface
B670	670/30 (655LP)	CD8	PerCP Cy5.5	RPA T8	Biolegend	
V650	670/30 (655LP)	CCR7	BV650	G043H7	Biolegend	
G780	780/60 (750LP)	CD45RA	PeCy7	HI100	Biolegend	
B530	530/30 (505LP)	CD57	BB515	NK-1	BD	
G586	586/15 (550LP)	PD1	PE			
V450	450/50	TIGIT	BV421	741182	BD	
G610	610/20 (595LP)	EOMES	PE-eFluor 610	WD1928	eBiosciences	Intracellular
V785	780/60 (750LP)	T-bet	BV785	4B10	Biolegend	
R670	670/30	IFNγ	APC	B27	BD	
